# Comparative Evaluation of Clinical and Radiographic Success of Formocresol, Propolis, Turmeric Gel, and Calcium Hydroxide on Pulpotomized Primary Molars: A Preliminary Study

**DOI:** 10.5005/jp-journals-10005-1400

**Published:** 2016-12-05

**Authors:** Shivayogi M Hugar, Pratibha Kukreja, Shweta S Hugar, Niraj Gokhale, Harsha Assudani

**Affiliations:** 1 Reader, Department of Pediatric and Preventive Dentistry, KLE VK Institute of Dental Sciences, Belgaum, Karnataka, India; 2 Postgraduate Student, Department of Pediatric and Preventive Dentistry, KLE VK Institute of Dental Sciences, Belgaum, Karnataka, India; 3 Senior Lecturer, Department of Periodontics, KLE VK Institute of Dental Sciences, Belgaum, Karnataka, India; 4 Senior Lecturer, Department of Pediatric and Preventive Dentistry, KLE VK Institute of Dental Sciences, Belgaum, Karnataka, India; 5 Postgraduate Student, Department of Pediatric and Preventive Dentistry, KLE VK Institute of Dental Sciences, Belgaum, Karnataka, India

**Keywords:** Formocresol, Propolis, Pulpotomy.

## Abstract

**Aims:**

Despite various advents in technology, the present era marks a shift to phytotherapeutics and alternative modalities to conventional endodontic treatments. Newer endodontic modalities have been developed inculcating the ancient system of medicine. The present study was done to compare and evaluate the clinical pulp response and radiographic signs after pulpotomy in four groups of primary molar teeth treated with formocresol (control), propolis extract, turmeric gel, and calcium hydroxide respectively.

**Materials and methods:**

Following ethical clearance, 90 primary molar teeth in 45 pediatric patients, aged between 4 and 9 years, were selected for pulpotomy. These were then randomly divided by split-mouth technique into two groups as experimental (propolis extract/turmeric gel/calcium hydroxide) and control (formocresol) groups. The patients were followed up for 6 months for clinical and radiographic signs and symptoms to evaluate the success of treatment.

**Results:**

A comparable clinical and radiographic success rate was seen with all experimental groups as compared to the control (formocresol) group.

**Conclusion:**

With concerns about the safety of formocresol appearing in the dental and medical literature for more than 20 years, the materials used in this study can be considered as promising alternatives for formocresol in pediatric endodontic treatment.

**How to cite this article:**

Hugar SM, Kukreja P, Hugar SS, Gokhale N, Assudani H. Comparative Evaluation of Clinical and Radiographic Success of Formocresol, Propolis, Turmeric Gel, and Calcium Hydroxide on Pulpotomized Primary Molars: A Preliminary Study. Int J Clin Pediatr Dent 2017;10(1):18-23.

## INTRODUCTION

The ultimate aim of vital pulp therapy is the treatment of reversible pulpal injuries in primary and permanent teeth, maintaining the vitality and function of dental pulp. Vital pulp therapy includes two therapeutic approaches; i.e., indirect pulp treatment in cases of deep dentinal cavities and direct pulp capping or pulpotomy in cases of pulp exposure.^1^ No area of treatment in pediatric dentistry has been more controversial than vital pulp therapy, which has been debated for decades now.^2^ Formocresol, first introduced by Buckley in 1904, has long been considered the “gold standard” to which all other medicaments have been compared for pulpotomies in primary teeth. It is preferred because of its excellent bacteriostatic and fixative properties, and it has a success rate ranging from 55 to 98%.^3^

But, many concerns have been raised over the use of formocresol, mainly due to its toxicity and potential carcinogenicity.^1^ The constituents of formocresol, formaldehyde (a mutagen and carcinogen), and cresol (a caustic agent) still raise concerns among dentists and patients, even though its systemic distribution has resulted in no documented ill effects.^3^ Also, the International Agency for Research on Cancer (IARC) has classified formaldehyde as carcinogenic to humans in June 2004, leaving the profession to look for other viable alternatives to formocresol.^4^

Several pulp dressing medicaments have been proposed to maintain radicular pulp vitality that are equal to, if not better than, formocresol and can be used as alternatives to pulpotomies in primary teeth. These include electrosurgery, laser, glutaraldehyde, calcium hydroxide, freeze-dried bone, mineral trioxide aggregate, and sodium hypohlorite.^1,5,6^

Hence, the purpose of the present study was to evaluate and compare the clinical pulp response and radio-graphic signs after pulpotomy in four groups of primary molar teeth treated with formocresol (control), propolis extract, turmeric gel, and calcium hydroxide respectively.

## MATERIALS AND METHODS

The ethical clearance for the study was obtained from institutional ethical board, KLE VK Institute of Dental Sciences, Belgaum, Karnataka, India. About 90 primary molar teeth in 45 pediatric patients aged between 4 and 9 years were selected based on the criteria^7^ as:

**Fig. 1:**
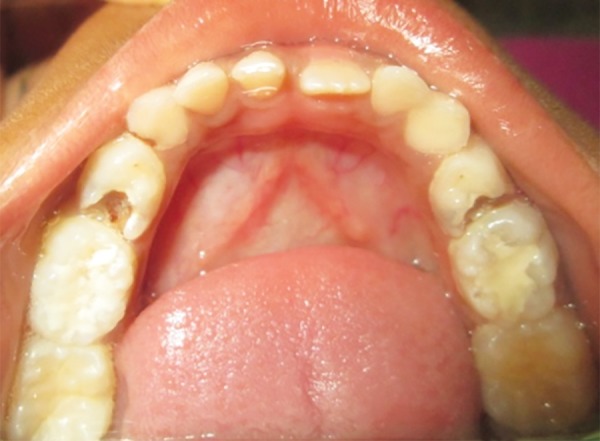
Case selection for pulpotomy: Clinical criteria

- Exposure of vital pulp due to dental caries, approximating to the pulp radiographically.
- Absence of symptoms indicative of advanced pulpal inflammation, such as spontaneous pain or history of nocturnal pain.
- No clinical and radiographical evidence of pulp degeneration, such as excessive bleeding from the root canal, tenderness to percussion, swelling or sinus tract, mobility, internal resorption, interradicular, and/or periapical bone destruction, advanced physiological root resorption.
- Teeth should be restorable after completion of the procedure.

**Fig. 2:**
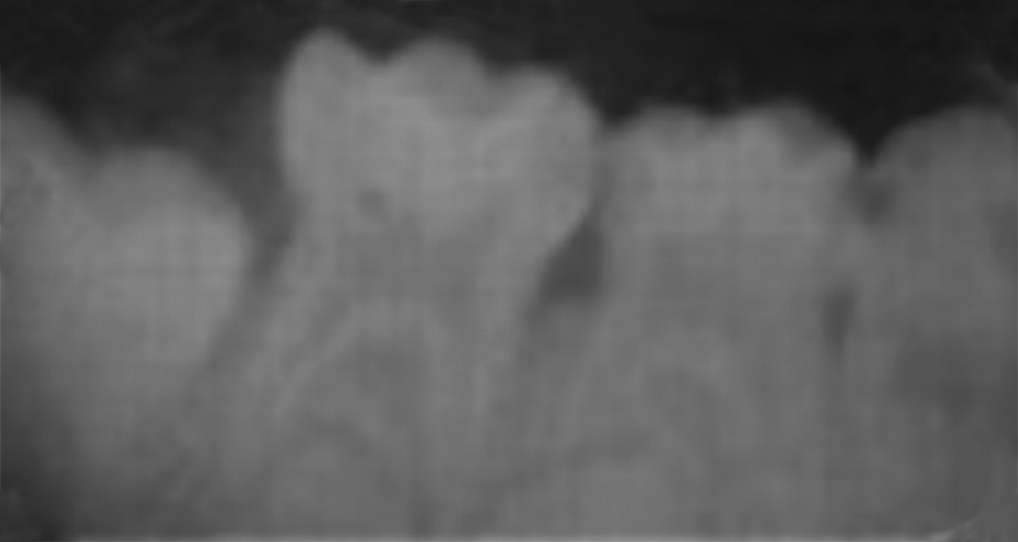
Case selection for pulpotomy: Radiographic criteria

Patients with any systemic diseases, dentofacial anomalies, medical history, and nonrestorable teeth were excluded from the study. The teeth indicated for pulpotomy were assessed by a single caliberated clinician who also performed the procedures/techniques and evaluated after 12 weeks. The teeth under study in case of selected children were chosen who required minimum two pulpotomies in either arch or same arch preferably each on the opposite side (i.e., right and left) (Figs 1 and 2). The teeth on the right side were assigned to the formocresol (control) group in all the patients. The teeth on the left side for 15 patients were assigned each for propolis (experimental group I), turmeric (experimental group II), and light-cured calcium hydroxide (experimental group III) respectively.

### Technique for Pulpotomy

**Fig. 3:**
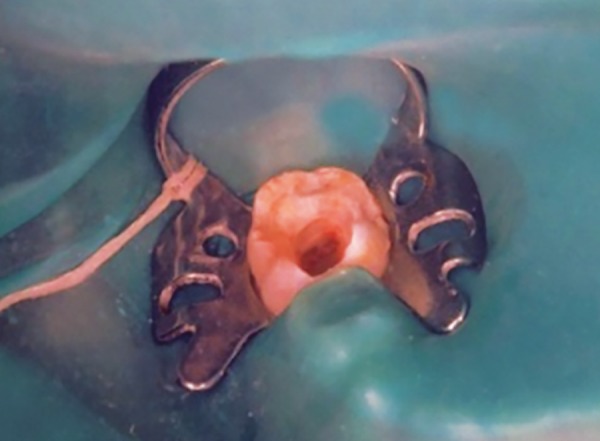
Technique for pulpotomy

The pulpotomy procedure was performed under local anesthesia rubber dam isolation. Following the establishment of cavity outline form, all peripheral caries was removed before the pulp was exposed. The coronal pulp was removed with a sharp spoon excavator. The pulp was amputated at the entrance of the root canals, and the pulp chamber was irrigated with normal saline to prevent dentin chips from being forced into the radicular pulp. Following irrigation, sterile cotton pellets were used and applied to the amputated pulp stumps to aid hemostasis (Fig. 3). Depending upon the group assigned, the amputation site was treated as follows:

### Control Group

A cotton pellet dampened (duly blotted and virtually dry) with a 1:5 dilution of formocresol (Trisol, Vishal Dentocare Pvt. Ltd., Ahmedabad, Gujarat, India) was applied to the amputation site for 5 minutes and removed. A thick mix of zinc oxide eugenol cement was placed into the coronal pulp chamber. A layer of intermediate restorative material (IRM) was placed in the same appointment.

### Experimental Group I

A cotton pellet moistened (after removing excess) with 33% green propolis extract (water soluble Korean) was applied to amputation site for 5 minutes and removed. A thick mix of zinc oxide eugenol cement was placed into the coronal pulp chamber. A layer of IRM was placed in the same appointment.

**Fig. 4:**
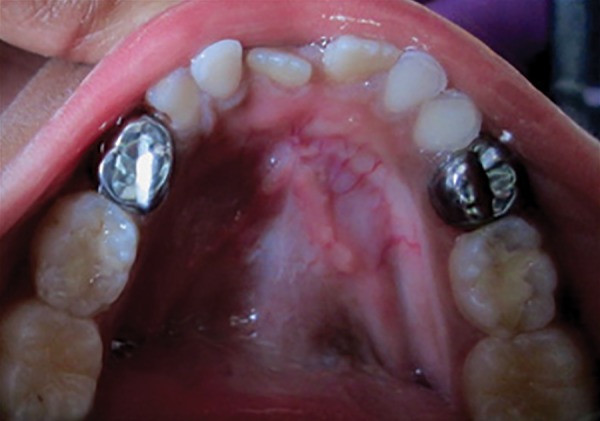
Completed pulpectomy procedure

### Experimental Group II

Prepared turmeric *(Curcumin longa)* gel was placed directly on the amputation site for 5 minutes and a thick mix of zinc oxide eugenol cement was placed into the coronal pulp chamber. A layer of IRM was placed in the same appointment.

### Experimental Group III

The amputation site was covered with light-cured calcium hydroxide and cured according manufacturer’s instructions. The remaining pulp chamber was filled with resin modified glass ionomer cement (GIC).

A confirmatory radiograph was taken (Fig. 4), and after 7 days, in the absence of any clinical symptoms, teeth were restored with stainless steel crowns (3M ESPE) cemented with luting (type I) GIC. The evaluation of the pulpotomized teeth at follow-up visits was done at 1, 3, and 6 months respectively by two standardized and calibrated examiners who were double-blinded. The patients were evaluated clinically and radiographically according to the following criteria^3^:

- *Clinical criteria:* Teeth were evaluated for the presence of any pain, soft tissue swelling, mobility, and other signs of clinical disease.
- *Radiographic criteria:* Intraoral periapical/bitewing radiographs were taken at the recall appointments and were evaluated for the presence of internal or external resorption, interradicular or periapical bone loss, or widening of periodontal ligament space.

The absence or presence of these signs and symptoms at the end of 1, 3, and 6 months were used for determination of cumulative success and failure of teeth respectively. All the data were collected and statistically analyzed using the Statistical Package for the Social Sciences software version 19.0.

**Fig. 5:**
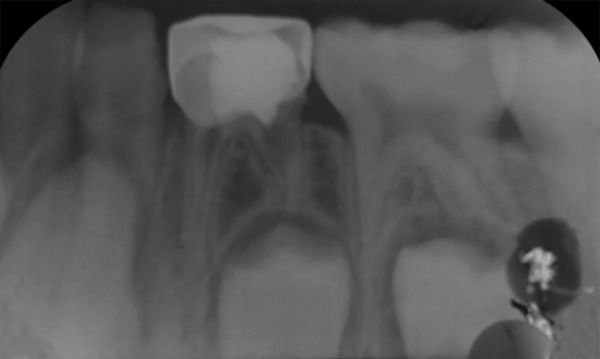
Radiographic failure in the form of internal resorption seen with turmeric pulpotomy

**Fig. 6:**
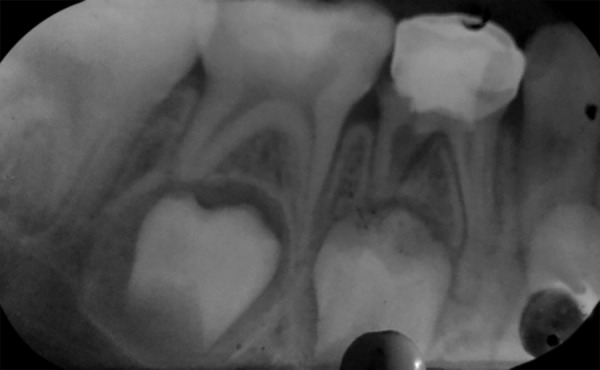
Radiographic failure in the form of internal resorption seen with calcium hydroxide pulpotomy

### RESULTS

**Graph 1:**
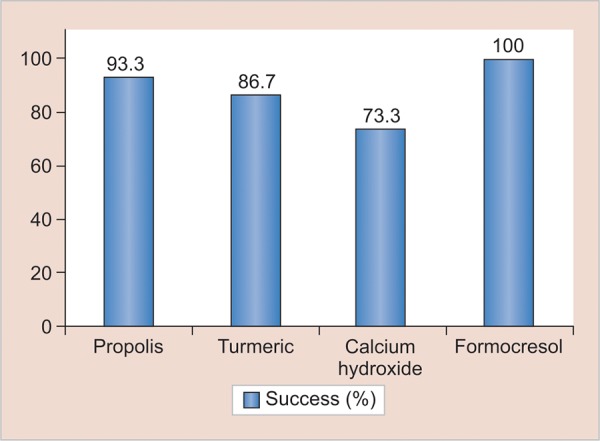
Success percentage assessed by radiographic findings

**Graph 2:**
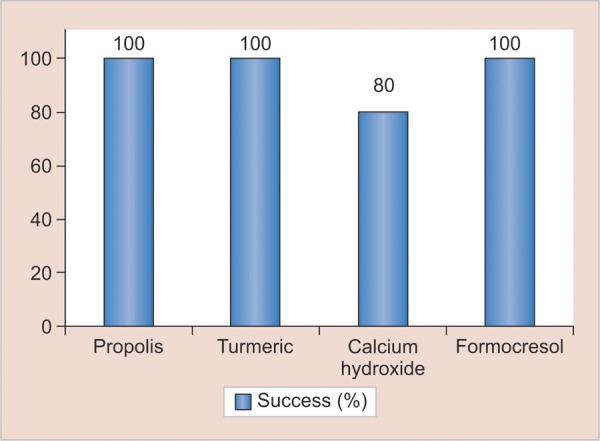
Success percentage assessed by clinical signs and symptoms

At the end of 1 and 3 months, teeth in all groups were judged to be clinically and radiographically sound as perceived by absence of clinical signs and symptoms and intraoral radiographs. However, at the end of 6 months, one tooth in the experimental group I (propolis) exhibited signs of radiographic failure but was clinically judged to be free of symptoms. Similarly, failures in the form of internal resorption was seen in two teeth of experimental group II (turmeric) (Fig. 5) which too were free of any clinical signs and symptoms. Conversely, in experimental group IV (calcium hydroxide), four teeth were found to be failures, as observed on radiographs (Fig. 6). Out of these, three teeth were accompanied with clinical symptoms of pain (two cases) and abscess (one case) (Table 1, Graphs 1 and 2). All the teeth from the control group (formocresol) were judged to be clinically and radiographically sound at the end of 1, 3, and 6 months.

**Table 1:** **Table 1:** Success and failure rate of propolis, turmeric, calcium hydroxide, and formocresol pulpotomies (Note: There is no statistical test possible as formocresol has no failure rate, which means all are zero for failure. So, only percentages can be expressed.)

				*Radiographic*				*Clinical*			
*Groups*				*Frequency*		*Percentage*		*Frequency*		*Percentage*	
Propolis (experiment I)		Failure		1		6.7		0		0	
		Success		14		93.3		15		100	
Turmeric gel (experiment II)		Failure		2		13.3		0		0	
		Success		13		86.7		15		100	
Calcium hydroxide (experiment III)		Failure		4		26.7		3		20	
		Success		11		73.3		12		80	
Formocresol (control)		Failure		0		0		0		0	
		Success		45		100		45		100	

**Table 2:** **Table 2:** Comparison between success rates of propolis and turmeric gel

				*Clinical*				*Radiographic*			
*Propolis vs turmeric gel*				*Frequency*		*%*		*Frequency*		*%*	
Propolis		Success		15		100		14		93.3	
		Failure		0		0		1		6.7	
Turmeric		Success		15		100		13		86.7	
		Failure		0		0		2		13.3	
Fisher’s exact test				Not applicable				p-value = 0.612*			

*Not statistically significant

**Table 3:** **Table 3:** Comparison between success rates of propolis and calcium hydroxide

				*Clinical*				*Radiographic*			
*Propolis vs calcium hydroxide*				*Frequency*		*%*		*Frequency*		*%*	
Propolis		Success		15		100		14		93.3	
		Failure		0		0		1		6.7	
Calcium hydroxide		Failure		3		20		4		26.7	
		Success		12		80		11		73.3	
Fisher’s exact test				p-value = 0.222*				p-value = 0.329*			

*Not statistically significant

**Table 4:** **Table 4:** Comparison between success rates of turmeric and calcium hydroxide

				*Clinical*				*Radiographic*			
*Turmeric vs calcium hydroxide*				*Frequency*		*%*		*Frequency*		*%*	
Turmeric		Success		15		100		13		86.7	
		Failure		0		0		2		13.3	
Calcium hydroxide		Failure		3		20		4		26.7	
		Success		12		80		11		73.3	
Fisher’s exact test				p-value = 0.224*				p-value = 0.653*			

*Not statistically significant

The comparison between the success rates of the various test materials was done using Fisher’s exact test. No statistically significant difference was found in the clinical and radiographic success rates of the various test materials when compared individually (Tables 2 to 4).

## DISCUSSION

There is overwhelming concern worldwide about the risk of environmental mutagens and carcinogens, like formaldehyde, to children. The increase in cancer has been linked to mutagenic and carcinogenic agents. Since June 2004, the IARC has reclassified formaldehyde as a known human carcinogen. Recently, formaldehyde has been strongly associated with leukemia while generally accepted as a direct cause of nasopharyngeal cancer.

Despite the hundreds of articles that have supported the mutagenicity (genotoxicity), carcinogenicity and toxicity of formaldehyde, it is still used today in full strength by an alarming number of clinicians around the world.^8^

The foundation of the argument against the use of formocresol in dentistry is the belief that, upon placement in the pulp chamber, unmetabolized formocresol (primarily formaldehyde) becomes systemically distributed. Free formaldehyde present in the circulation could react with macromolecules, thereby potentially causing mutagenic and/or cytotoxic changes in muscle, liver, kidney, heart, spleen, and lung tissue.^9^

Owing to these concerns about the use of formocresol, various alternatives have been tried and tested over time. Calcium hydroxide had been proposed as a strong proponent for the replacement of formocresol for vital pulp therapy in children since its introduction. However, clinical studies have proposed that complete and incomplete dentin bridges are formed in amputated pulps beneath calcium hydroxide. The results of the present study are in accordance with previous investigations done by Waterhouse et al,^7^ Magnusson et al, Markovik et al,^10^ Huth et al,^11^ and Zurn et al.^3^ All these studies have reported inferior clinical and radiographic success rate of calcium hydroxide in comparison to formocresol over varying periods of time.^1^

All the earlier studies used the paste/powder liquid form of calcium hydroxide. Evidence from Waterhouse et al concluded that calcium hydroxide in its pure powder form was a clinically acceptable alternative to formocresol. The most frequently reported side effect with calcium hydroxide use in primary teeth is internal resorption. This phenomenon, as suggested by Heilig et al,^12^ occurs owing to the “embolization” process by which particles of calcium hydroxide induce focal points of inflammation into the pulp tissue.^3^ This was attempted to overcome by using a hard-setting calcium hydroxide, and the success rate was found to be 88% over a 9-month follow-up period. Another attempt to reduce emboliza-tion was made with the use of visible light-cured calcium hydroxide, as it was thought that the resin material may stabilize the compound limiting its breakdown. However, no improvement in clinical and radiographic findings was observed by Zurn et al^3^ with the use of visible light-cured calcium hydroxide; similar results were observed in the present study.

Propolis is reputed to have antiseptic, antibacterial, antimycotic, astringent, spasmolytic, anti-inflammatory, anesthetic, antioxidant, antitumoural, antifungal, anti-ulcer, anticancer, and immunomodulatory effects. It has been used in a variety of applications, which include ointments and creams used in wound healing, treatment of burns, skin problems, and ulcers.

It has been known for a long time that propolis and its extracts have a positive effect on tissue regeneration. It is proved that alcoholic solution of propolis accelerates the tissue regeneration process, promotes the healing processes in damaged cartilage, and enhances ossification in artificially induced bone defects. Also, ethanolic extract of propolis supports regeneration of dental pulp and reduces inflammatory and degenerative processes as well.^13^

There is a limited amount of evidence for the use of propolis in pediatric endodontic therapy, and most investigations in the past have focused on using propolis as a pulp-capping material in animal models. It was found that, in addition to having a low irritating potential, propolis induces a healing process in both epithelial and pulp tissue with formation of collagen and dentin bridges.^14^ Excellent clinical pulp response has been found with the use of propolis as a pulp-capping material in the present study. However, the radiographic failure may be attributed to disintegration of propolis compounds inducing focal areas of inflammation into the vital radicular pulp tissue causing internal resorption similar to that of calcium hydroxide.

*Curcumin longa,* also called as turmeric, contains three curcuminoids: Curcumin as well as volatile oils (tumer-one, atlantone, and zingiberone), sugars, proteins, and resins. It has a wide range of pharmacological applications, owing to its antioxidant, anti-inflammatory, and antimicrobial properties.^15^ Limited evidence is available on the pulpal response to curcumin formulations. In the present study, acceptable clinical and radiographic results were obtained with the use of turmeric as a pulpotomy agent in primary molars. Long-term histologic analysis is recommended to account for the failures that occurred in the present study with the use of turmeric.

## CONCLUSION

As the controversy regarding the use of formocresol in vital pulp therapy of primary teeth is never-ending, its replacement with another material has become the need of the hour. With the combination of traditional medicine and the advent of technology, more phytotherapeutic materials are underway for finding the next gold standard material in pediatric endodontics. Excellent clinical and acceptable radiographic success was observed with the test materials. This study paves ways for further long-term studies to test the performance of the test materials used.
